# Zika virus infection in pregnant women in Honduras: study protocol

**DOI:** 10.1186/s12978-016-0200-6

**Published:** 2016-07-16

**Authors:** Pierre Buekens, Jackeline Alger, Fernando Althabe, Eduardo Bergel, Amanda M. Berrueta, Carolina Bustillo, Maria-Luisa Cafferata, Emily Harville, Karla Rosales, Dawn M. Wesson, Concepcion Zuniga, for the ZIPH Working Group, Pierre Buekens, Jackeline Alger, Fernando Althabe, Eduardo Bergel, Amanda M. Berrueta, Carolina Bustillo, Maria Luisa Cafferata, Emily Harville, Jorge Largaespada, Ivette Lorenzana de Rivera, Carlos Matta, Karla Pastrana, Karla Rosales, Dawn Wesson, Concepción Zúniga

**Affiliations:** School of Public Health and Tropical Medicine, Tulane University, 1440 Canal Street, Ste. 2430, New Orleans, LA 70112 USA; Instituto de Enfermedades Infecciosas y Parasitología Antonio Vidal; Hospital Escuela Universitario, Facultad de Ciencias Médicas, UNAH, Calle La Salud, Boulevard a Suyapa, Colonia Quezada, Tegucigalpa, Honduras; Instituto de Efectividad Clínica y Sanitaria (IECS), Dr. Emilio Ravignani 2024, C1414CPV Buenos Aires, Argentina; Departamento de Ginecología y Obstetricia, Hospital Escuela, Calle La Salud, Boulevard a Suyapa, Colonia Quezada, Tegucigalpa, Honduras; Unidad de Investigación Clínica y Epidemiológica Montevideo (UNICEM), Hospital de Clinicas, Avenida Italia s/n – piso 4°, Montevideo, 11600 Uruguay; Department of Epidemiology, Tulane School of Public Health and Tropical Medicine, Tulane University, Ste. 2012, 1440 Canal St., New Orleans, LA 70112 USA; Región Sanitaria Metropolitana Distrito Central de Francisco Morazán, Tegucigalpa, Honduras; School of Public Health and Tropical Medicine, Tulane University, 1324 Tulane Avenue, JBJ Building, Room 509, New Orleans, LA 70112 USA; Instituto de Enfermedades Infecciosas y Parasitología Antonio Vidal; Hospital Escuela Universitario, Calle La Salud, Boulevard a Suyapa, Colonia Quezada, Tegucigalpa, Honduras

**Keywords:** Honduras, Microcephaly, Pregnancy, Zika virus

## Abstract

**Background:**

Although there is increasing evidence for a relationship between symptomatic Zika virus (ZIKV) maternal infection, and microcephaly, a firm causal relation has yet to be established by epidemiologic studies. Studies also need to be conducted in recently infected settings. Our objectives are to assess the frequency of ZIKV infection during pregnancy in Honduras and the association of microcephaly with ZIKV infection.

**Methods/Design:**

We will perform a prospective study enrolling pregnant women at their first antenatal visit and following them up until delivery. At the time of enrollment, women will be interviewed to collect socio-demographic data, data needed to locate them for potential additional follow-up, and data about ZIKV symptoms during pregnancy. We will also collect maternal blood as soon as possible after enrollment. A probable maternal ZIKV infection will be defined as positive for maternal ZIKV IgM. A confirmed maternal ZIKV infection will be defined as positive for ZIKV IgM confirmed by plaque reduction neutralization test. Microcephaly at birth will be defined as an occipito-frontal circumference <2SD for sex and gestational age. Our objective is to enroll 2000 pregnant women. In a first step, we will follow a case cohort design and only analyze blood samples for cases and a sub-cohort of 200 women randomly selected. Blood samples for the entire population will be analyzed at a later stage if funds are available.

**Discussion:**

This protocol was designed to be implemented with minimal resources. It allows a cohort to be built, which could be a foundation for future in-depth and follow-up studies.

## Plain english summary

There is increasing evidence that mothers infected with Zika virus (ZIKV) during pregnancy could have a baby with microcephaly (a small head), but doubts persist and the magnitude of this risk is still uncertain. Studies also need to be conducted in countries where ZIKV just arrived. Our objectives are to assess the frequency of ZIKV infection during pregnancy in Honduras and the association of microcephaly with ZIKV infection.

We will perform a study enrolling pregnant women at their first antenatal visit and following them up until delivery of their baby. At the time of first antenatal care visit, women will be asked questions about themselves, such as their level of education; about the best way to locate them for potential additional follow-up; and about ZIKV symptoms during pregnancy. We will also collect maternal blood as soon as possible after enrollment. We will ask the hospital where they will deliver to measure the head of the baby. Our objective is to enroll 2000 pregnant women. We will analyze the blood samples of the mothers of babies with microcephaly and of some of the mothers of normal babies. Blood samples of everybody will be analyzed at a later stage if funds are available.

## Background

Beginning in October 2015, Brazil reported a cluster of cases of microcephaly in newborns from the Northeast Region [1]. Additionally, French Polynesia reported an increased number of central nervous system malformations in neonates from March 2014 to May 2015 [1]. Although there is increasing evidence for a relationship with symptomatic Zika virus (ZIKV) maternal infection, a firm causal relation has yet to be established [2, 3]. ZIKV was detected in the amniotic fluid [4] and fetal brain tissue [5] of fetuses diagnosed with microcephaly antenatally and confirmed after birth. It has been also shown in in vitro experiments that ZIKV infects human cortical neural cell progenitors and limits their growth [6]. Data from French Polynesia suggest that first-trimester infections are linked to the highest risk of fetal complications [7]. A recently published study conducted in Rio de Janeiro, Brazil, reported that, within a cohort of pregnant women who had clinically symptomatic, laboratory-confirmed ZIKV infection during pregnancy, 29 % had fetal cranial-brain anomalies identified by ultrasound [3]. Women without ZIKV infection had no cases diagnosed antenatally, but the sample size was small. One of the difficulties of studies where cases are identified at birth is that it is often impossible to determine if a woman was infected by ZIKV earlier in pregnancy. The virus can be identified by RT-PCR only during a few days after infection, and IgM can only be identified for a few weeks after infection [8]. Another challenge is the serological cross-reactivity between ZIKV and other related viruses (e.g., dengue) [8]. A review of the evidence supporting a causal relationship between ZIKV and microcephaly noted that the “consistent findings by ≥2 high-quality epidemiologic studies” criterion was only partially met [9].

In response to this situation, the Emergency Committee convened by the World Health Organization (WHO) on March 8th recommended that research should be intensified, with particular attention to studies that are conducted in more recently infected settings [2]. In Honduras, 19,693 suspected cases of ZIKV infection have been reported during the first nineteen weeks of 2016 [10]. An earlier study performed in Tegucigalpa, Honduras, showed that the frequency of dengue started increasing around June 1st and was at its maximum in July [11]. We are assuming that ZIKV infection will follow a similar pattern.

We will conduct a prospective study in Honduras aiming to assess the frequency of ZIKV infection during pregnancy, and the association of ZIKV infection with microcephaly. The Specific Aims are the following:

### Specific aim 1

To measure the frequency of ZIKV infection in pregnancy.

### Specific aim 2

To measure the association between symptomatic and asymptomatic maternal ZIKV infection during pregnancy and microcephaly at birth.

#### Hypothesis

Mothers who were infected with ZIKV during pregnancy have an increased risk of microcephaly.

## Methods/Design

### Overview of study design

We will perform a prospective study enrolling pregnant women at their first antenatal visit and following them up until delivery. We will enroll pregnant women attending the Alonso Suazo Health Center (Tegucigalpa, Honduras) at their first antenatal visit. At the time of enrollment, women will be interviewed to collect socio-demographic data, data needed to locate them for potential additional follow-up (e.g., household location, municipality of residence, cell phone number), and data about ZIKV symptoms during pregnancy. We will also collect maternal blood as soon as possible after enrollment. A probable maternal ZIKV infection will be defined as positive for maternal ZIKV IgM [2]. Head circumference at birth and gestational age estimated by last menstrual period and by Capurro scores [12] (or equivalent) will be used to diagnose microcephaly. Microcephaly at birth will be defined as an occipito-frontal circumference <2SD for sex and gestational age, using the INTERGROWTH-21st charts [13, 14]. In a first step, we will follow a case cohort design and only analyze blood samples for cases and a sample of the entire population (sub-cohort) [15]. Blood samples for the entire population will be analyzed at a later stage if funds are available.

### Settings

We will enroll women attending the Alonso Suazo Health Center (Tegucigalpa, Honduras), which had 7105 first antenatal visits in the last two years.

### Participants

#### Inclusion criteria

We will require written informed consent from the mother if she is 18 years or older. For mothers <18 years old, we will require written assent and written consent from at least one parent or guardian.

### Variables

#### Exposure and main outcome

- ZIKV infection - A suspected ZIKV infection during pregnancy will be defined as rash and/or fever and at least one of following signs or symptoms: arthralgia, arthritis, conjunctivitis (non-purulent/ hyperemic). A probable maternal ZIKV infection will be defined as positive for maternal ZIKV IgM. A confirmed maternal ZIKV infection will be defined as positive for ZIKV IgM confirmed by plaque reduction neutralization test (PRNT). These definitions are based on interim case definitions proposed by WHO as of February 12, 2016, and might be updated in the future [1]. Date (day, month, year) of suspected, probable, and confirmed diagnosis will be registered.
- Head circumference (cm) - A confirmed case of microcephaly will be defined as an occipito-frontal circumference <2SD for sex and gestational age, using the INTERGROWTH-21st charts [13, 14].

#### Other birth outcomes

- A live birth is a baby born with any sign of life, irrespective of gestational age [16].
- A stillbirth is a baby born without any sign of life and birthweight of ≥500 g; or, if missing, ≥22 completed weeks of gestation; or, if missing, body length ≥25 cm [16].
- Gestational age (completed weeks), based on the Last Menstrual Period (LMP) or a clinical estimate. The clinical estimate will be based on the Capurro Score (or equivalent) [12]. This score is the standard method to evaluate gestational age by physical examination of an infant at birth, widely used in Latin America. The clinical estimate will be used if the discrepancy between the LMP-based estimate and the clinical estimate is greater than 10 days; a preterm birth is <37 weeks; a very preterm birth is <32 weeks.
- Multiple pregnancy.
- Birthweight (grams); a low birthweight baby weighs <2500 g; a very low birthweight baby weighs <1500 g.
- Body length (cm).

#### Maternal characteristics

- Maternal age (years); parity (including current birth).
- Reproductive history (year of occurrence): previous pregnancies, abortions, stillbirths, live births, infants’ or children’s death.
- Educational level (completed years).
- Household location and municipality.

### Data sources: clinical data, socio-demographic data, and geographical data

#### Clinical data and socio-demographic data

Data about the mother will be collected by interviewing the mother and from the medical histories. Data about the newborn will be collected from medical histories. An insertion tape (Seca 212 or equivalent) will be recommended for measuring head circumference and an infantometer (Seca 416 or equivalent) for length. Study personnel will be trained in abstracting medical histories.

#### Geographical data

Street addresses will often be unreliable in the areas where participating women live; thus, detailed information will be collected at enrollment:

- Household location (addresses will be registered if available).
- Municipality of residence. Interviewers will have a list of municipalities available during the interview and will verify that the name of the municipality is valid.
- Cell phone number of the participants.

Study personnel familiar with the area will use the information provided by the mother to geocode the household location using Google Earth. In a previous study, our staff were able to locate the households in the field in more than 90 % of the cases and used Google Earth to document the locations. If the probable household location is within the municipality of residence provided by the mother, the municipality will be confirmed as valid. In case of discrepancy, we will call the mother and request additional information.

### Data sources: blood

#### Blood samples

A minimum of 5 mL of venous maternal blood and no more than 10 mL will be collected as soon as possible after enrollment. We estimate from our previous studies that >99 % of the maternal samples will be usable.

#### Storage of biological samples

Consent forms will include an opt-out provision that otherwise allows participating study institutions to store blood specimens for up to ten years for confirmatory studies (including diagnosis of co-infections) and for potential genetic studies linked to mother-to-child transmission of ZIKV. In our previous studies, >99 % of the participating women have accepted the storage.

Blood samples will be stored in part on filter paper kept in plastic bags with desiccant at 4 °C until use, and additional samples (e.g., serum or plasma) will be frozen at or below −20 °C until use. Freezer-safe barcode labels (Brady, Milwaukee, WI, or equivalent) will be pasted on each of the biological samples and will be scanned to document the location of the samples. Temperatures of freezers and refrigerators will be monitored by digital temperature data loggers (SM320, Dickson, Addison, IL, or equivalent). The Data Center will manage the barcode and temperature data.

#### Laboratory procedures

Investigators and staff performing the laboratory procedures will be blinded for the presence of microcephaly. We will measure ZIKV IgM on maternal blood, using tests and procedures recommended by the CDC [8]. Stored specimens will also allow for future serological testing, molecular analyses, and testing for insecticides, including larvicides.

#### Data management

Data management will be similar to the plan we have used in previously funded studies and will be coordinated by the Institute for Clinical Effectiveness and Health Policy (Instituto de Efectividad Clínica y Sanitaria [IECS], Buenos Aires, Argentina). Data will be collected on paper forms designed specifically for the study. For each participating woman, a study number (alpha-numeric, with check digit) will be assigned. A set of labels with each number will be designed to be pasted on the data forms of the mother and her newborn, and a freezer-safe barcode label (Brady, Milwaukee, WI, or equivalent) will be pasted on each of the biological samples. An inclusion form will be used to collect data during participants’ enrollment. Names and other personal identifiers will be recorded in the inclusion form header, which will be stripped from the main body containing data. Each part will be identified with a study label number. The inclusion form body will be sent to the local data center for data entry. This system ensures that personal identifiers will not be taken from the study site and will be kept securely by the site coordinator.

The data forms will be entered in OpenClinica, a secure web data management system (v 3.1.4.1or higher), or REDCap [17] which is an open source software for clinical research studies using distributed data entry. Additionally, digital pictures (with date and time) or scans of each data form will be taken and will be regularly sent (encrypted) to IECS. This system will allow for a digital backup of all study data forms, as well as a parallel second data entry of 10 % of the forms at IECS, to detect systematic errors at data entry.

In addition, the Data Center will manage the information from the freezers’ and refrigerators’ temperature data loggers, preparing graphs and detecting values out of expected range. The Data Center will also manage the stock information from the scanned biological samples barcodes.

Monitoring will be done through weekly on-site visits to the Alonso Suazo Health Center by the site coordinator, who will complete weekly reports that will be submitted to the Data Center. Data Center staff will conduct weekly monitoring calls with the site coordinator to discuss site reports and will produce weekly summary reports, which will be submitted to the Principal Investigator and the study coordinating center. For quality assurance, the Principal Investigator and Data Center staff will perform at least one site visit. During the site visits, source data and biological samples verification will be performed in 10 % of cases and 10 % of controls. All storage and laboratory procedures will be audited as well.

### Sample size

Our objective is to enroll 2000 pregnant women. A sub-cohort of 200 women will be randomly selected.

#### Specific aim 1

The sub-cohort of 200 women will allow us to measure a seroprevalence of 5 % with a 95 % confidence interval of + − 3 %.

#### Specific aim 2

**Fig. 1:**
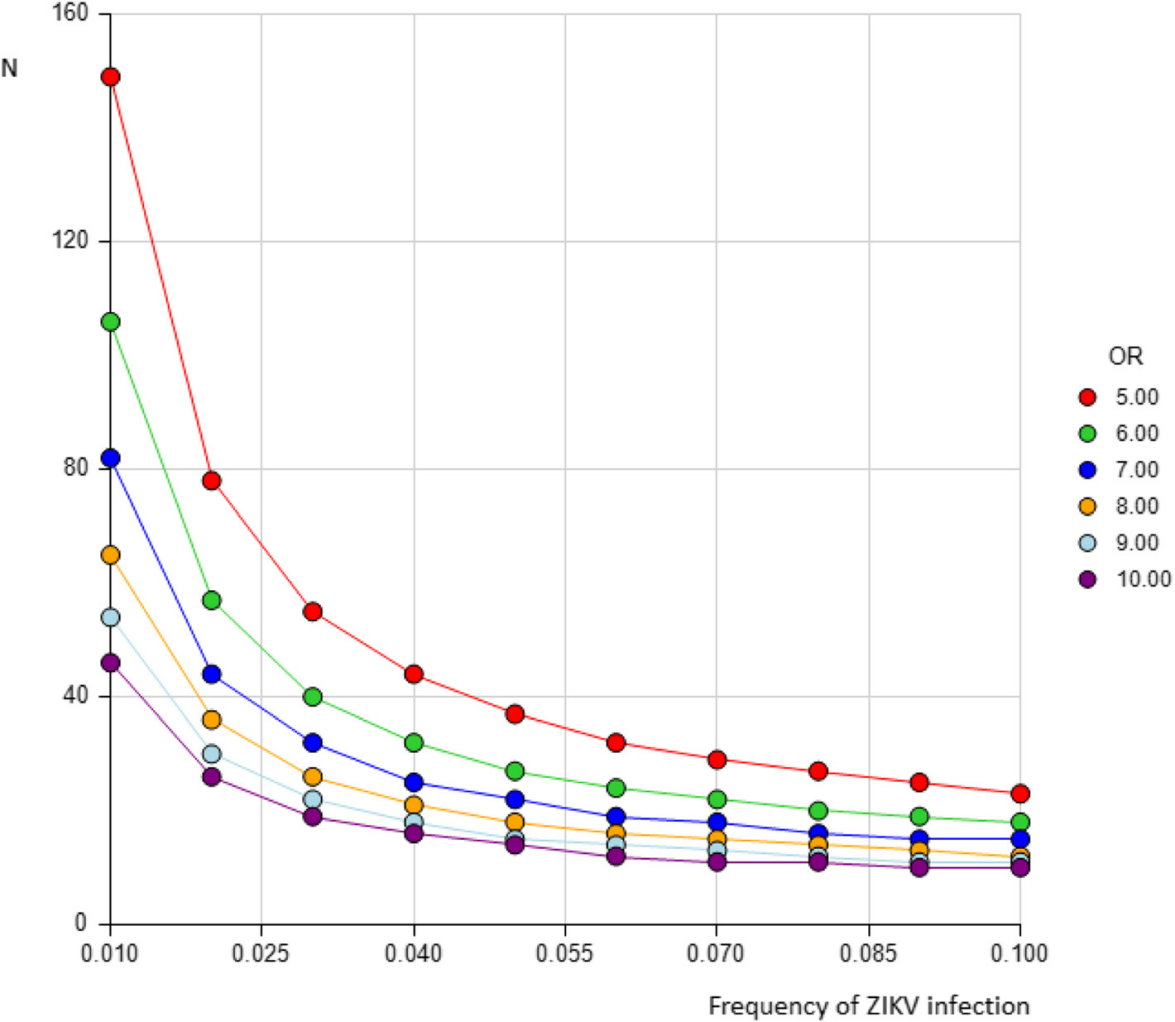
Number of cases (N) needed by frequency of ZIKV infection and odds ratio (OR) of the association with microcephaly (3 controls, power 0.8, alpha 0.05, not matched)

Assuming a 2 % frequency of microcephaly, we estimate that 40 cases and a sub-cohort of 200 women will give us 80 % power (0.05 alpha level) to detect an odds ratio (OR) of 5 for the association between ZIKV infection and microcephaly, if the prevalence of ZIKV infection is 5 % (Fig. 1). A higher prevalence of ZIKV infection would increase the power.

### Plan for data analysis

We will initially follow a case-cohort design. A sub-cohort of 200 women will be randomly selected. We will compute the frequency of ZIKV infection among the sub-cohort (Specific Aim 1). Blood samples for the entire population will be analyzed at a later stage if funds are available, and frequency of ZIKV infection will be computed accordingly. Logistic regression analysis will be used to estimate crude and adjusted ORs and corresponding 95 % Confidence Intervals (CIs) for the association between maternal ZIKV virus infection and microcephaly (Specific Aim 2). Potential confounders (e.g., municipality of residence, educational level) will be entered in the model. If the OR estimate changes by 10 % or more after controlling for a given variable, subsequent OR estimates will be adjusted for that variable. We will perform sub-group analysis by presence or absence of ZIKV symptoms (“Suspected ZIKV”) at or before the first antenatal visit.

We will use STATA v12.0 or higher (StataCorp, College Station, TX) for data analysis.

### Study organization

Our steering committee includes the Principal Investigator, the Co-Principal Investigator from Honduras, and one Co-Investigator from the Data Center. A local coordinating center will be established in Honduras. The IECS in Buenos Aires, Argentina, will act as the study Data Center, thereby providing a specific data monitoring system, assuring maintenance of high quality databases, supervising all data collection procedures, and arranging for the most efficient transfer of study data.

### Timeline and tasks

The study will include two months of preparatory activities, an estimated 12 months of enrollment, and 10 months for follow up, laboratory procedures, data analysis, and reporting.

During the preparatory phase (I), we will develop (in Spanish) the Manual of Operations, the Data Management System, and data collection forms and post them on our password-protected website. We will also train the data collection personnel. During the enrollment phase (II), we will enroll women and perform lab analyses, which will continue during the last phase of the study (Phase III). Data collection at birth will continue during Phase III. Data analysis will be conducted during Phase III.

## Discussion

This protocol was designed to be implemented with minimal resources, with blood samples collected as early as possible during pregnancy being stored for future analyses. We are following a modular approach, with the current protocol being the foundation module on which additional future in-depth studies could be added. Potential future modules could include more in-depth data collection during pregnancy and follow-up studies of ZIKV infected mothers and their children.

## Abbreviations

IECS: Instituto de Efectividad Clínica y Sanitaria; IRB: Institutional Review Board; PRNT: plaque reduction neutralization test; WHO: World Health Organization; ZIKV: Zika Virus; ZIPH: Zika Virus in Pregnancy in Honduras
